# Cholesterol Acceptors Regulate the Lipidome of Macrophage Foam Cells

**DOI:** 10.3390/ijms20153784

**Published:** 2019-08-02

**Authors:** Antoni Paul, Todd A. Lydic, Ryan Hogan, Young-Hwa Goo

**Affiliations:** 1 Department of Molecular and Cellular Physiology, Albany Medical College, Albany, NY 12208, USA; 2 Department of Physiology, Michigan State University, East Lansing, MI 48824, USA

**Keywords:** ABCA1, ABCG1, cholesterol, macrophage, foam cells, atherosclerosis, LDL, lipidome, lipidomics, mass-spectrometry, oxysterols, reverse cholesterol transport

## Abstract

Arterial foam cells are central players of atherogenesis. Cholesterol acceptors, apolipoprotein A-I (apoA-I) and high-density lipoprotein (HDL), take up cholesterol and phospholipids effluxed from foam cells into the circulation. Due to the high abundance of cholesterol in foam cells, most previous studies focused on apoA-I/HDL-mediated free cholesterol (FC) transport. However, recent lipidomics of human atherosclerotic plaques also identified that oxidized sterols (oxysterols) and non-sterol lipid species accumulate as atherogenesis progresses. While it is known that these lipids regulate expression of pro-inflammatory genes linked to plaque instability, how cholesterol acceptors impact the foam cell lipidome, particularly oxysterols and non-sterol lipids, remains unexplored. Using lipidomics analyses, we found cholesterol acceptors remodel foam cell lipidomes. Lipid subclass analyses revealed various oxysterols, sphingomyelins, and ceramides, species uniquely enriched in human plaques were significantly reduced by cholesterol acceptors, especially by apoA-I. These results indicate that the function of lipid-poor apoA-I is not limited to the efflux of cholesterol and phospholipids but suggest that apoA-I serves as a major regulator of the foam cell lipidome and might play an important role in reducing multiple lipid species involved in the pathogenesis of atherosclerosis.

## 1. Introduction

Atherosclerosis represents one of the most complex and challenging disorders in industrialized societies. It is characterized by the buildup of lipid-filled plaques in the arterial walls in tandem with increased inflammation, necrosis, and fibrosis [1,2]. Along with meta-analysis of human population studies, recent genome-wide association studies (GWAS) in humans support higher levels of cholesterol-rich apolipoprotein-B containing lipoproteins (apoB-LPs) in the plasma increases the risk of coronary artery disease (CAD) [3,4,5]. As reviewed by Khera et al., GWAS studies identified that dysfunctional mutations in the genes involved in lipoprotein metabolism coincide with elevated plasma low-density lipoprotein (LDL) cholesterol, lipoprotein (a), and triglyceride-rich lipoproteins, leading to a significant increase in CAD casualties [5]. In addition, GWAS studies also identified non-lipid mediated CAD risk loci where the mutations of genes involved in vascular endothelial cell repair, smooth muscle cell proliferation, migration, and remodeling also increases the risk of CAD without abnormal elevation of the plasma apoB-LPs [5,6,7].

Lipid accumulation in the vascular wall is initiated by subendothelial retention of circulating apoB-LPs such as LDL. LDL is oxidized in both circulation and atherosclerotic lesions. Circulating oxidized LDL (oxLDL) has a higher affinity for vascular endothelial cells, and it is more susceptible to internalization by macrophages within the lesions [8,9,10]. Leukocyte and non-leukocyte derived macrophages in the arterial wall take up LDLs, becoming foam cells—a distinctive trait of atherosclerotic plaques [2,11,12,13]. Once oxLDL is internalized by macrophages via scavenger receptors, cholesterol ester (CE), a major lipid in the core of LDL, is hydrolyzed into FC in the lysosome. The FC is either effluxed out by diffusion or ATP-binding cassette transporter A1 (ABCA1) to the lipid-poor cholesterol acceptor, apoA-I, creating a nascent HDL particle. Subsequently, HDL-mediated FC efflux is accomplished via ATP-binding cassette subfamily member G1 (ABCG1). Surplus intracellular FC is re-esterified into CE by acyl coenzyme A: cholesterol acyltransferase-1 (ACAT1) and stored within cytosolic lipid droplets (LDs), a process to reduce cytotoxicity of FC [2,14,15,16,17].

The equilibrium of lipid quantity and composition in an atheroma depends on lipid influx carried by LDL and efflux to apoA-I and HDL. Reverse cholesterol transport (RCT), cholesterol removal from peripheral tissues (e.g., foam cells) to feces via either the hepatobiliary or transintestinal pathway is accomplished by cholesterol acceptors in both vascular and lymphatic circulation, most notably apoA-I and HDL [1,18,19,20,21,22]. Because RCT is a major route for the body to excrete cholesterols, activating RCT by increasing apoA-I levels in circulation reduces foam cell formation thereby preventing plaque buildup [23]. Athero-protective properties of apoA-I/HDL are also in part from their anti-inflammatory and anti-apoptotic functions [24]. However, certain modifications in the lipidome and the proteome of HDL lead to dysfunctions of HDL, rendering it pro-inflammatory and pro-atherogenic in some cases [25]. Thus, assessing RCT capacity along with lipid and protein compositions of HDL are considered more accurate cardiovascular disease (CVD) biomarkers than simply measuring the level of plasma HDL cholesterol [23,24,26,27,28].

Since cholesterol is enriched in LDL particles and human atherosclerotic plaques, many studies have been performed on cholesterol metabolism in atherosclerosis. However, recent lipidomic analyses of human plasma identified more than 500 lipid species and revealed that LDL alone contains more than 350 different lipid species [29,30]. Lipidomic analysis of human atherosclerotic plaques recently discovered plaque-enriched lipids including polyunsaturated CEs with long-chain fatty acids (FAs), lysophosphatidylcholines and certain sphingomyelin (SM) species [31]. Additionally, a comparison of plasma LDL and human plaque lipid composition identified potential lipid modifications in the plaques, and it is established that foam cells within the atherosclerotic microenvironment metabolize lipids in response to extracellular stimuli from other immune cells within the plaques which, overall, makes the lipidome of foam cells significantly different than that of circulating LDL particles [13,31]. For instance, oxysterols in human endarterectomy are much higher than in radial arteries and circulating LDL. Of note, certain oxysterols are much more bioactive and pro-inflammatory than cholesterol, and they are strongly correlated with CVD prevalence [32]. A supplement of oxysterols in cholesterol-rich diets promoted atherosclerosis in apolipoprotein E (apoE) and LDL receptor (LDLR) knockout (KO) mice [33,34]. Given this complexity, understanding the mechanism of eliminating these lipid species from the plaque might be a key to advance therapeutic strategies against atherosclerosis.

Although much research has investigated FC efflux regulated by extracellular cholesterol acceptors, the lipidome of oxLDL-loaded-foam cells and its remodeling by cholesterol acceptors remain poorly characterized. Therefore, we took advantage of lipidomics to profile over 500 lipid compounds of oxLDL-induced foam cells and to investigate how cholesterol acceptors remodel the entire foam cell lipidome beyond their effects on cholesterol removal. This study reveals that cholesterol acceptors not only reduce various oxysterols as well as FC, they also have a broad influence on the lipid profiles of foam cells including notable reductions of lipid species linked to atherosclerosis development.

## 2. Results

### 2.1. Highly-Oxidized LDL Loading Increases Intracellular Oxysterols Abundantly Found in Human Atheroma

**Figure 1:**
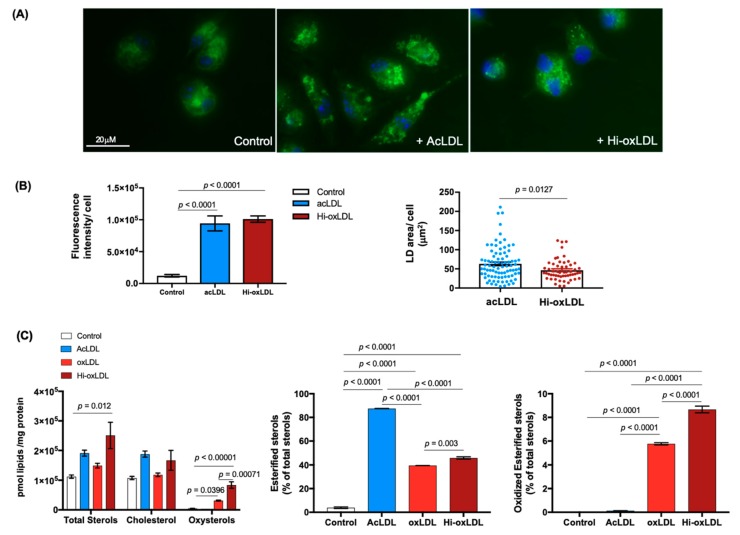
Oxidized low-density lipoprotein (LDL) enriches oxysterols in macrophage foam cells. (**A**) Foam cell formation. Mouse peritoneal macrophages (MPMs) plated on a coverslip remained untreated (left) or were loaded with either acLDL (middle) or hi-oxLDL (right) for 24 h to generate low-density lipoprotein (LDs). LDs were visualized with Bodipy 483/503 staining (green) and nuclei were stained with DAPI (blue). (**B**) Total Bodipy fluorescence intensity per cell represents total lipid contents (left). Area of LD per cell was measured and presented in μm^2^/cell. *n* = 88 for acLDL and *n* = 55 for Hi-oxLDL-treated MPMs. (**C**) Sterol contents of foam cells treated with modified LDLs. MPMs were treated with acLDL for 24 h, and oxLDL or hi-oxLDL for 48 h. Detected sterols using mass-spectrometry (MS) was normalized by mg protein and shown as pmol of lipids over μg of protein. The data were presented as mean ± standard error of mean (SEM).

In arteries, LDL particles retained beneath the endothelium undergo various modifications (e.g., oxidation) of the apolipoproteins and their lipids, and furthermore, these modified LDLs are favored and engulfed by lesional macrophages in an unfettered manner [10,35,36,37,38]. Sterols, a highly abundant lipid category in the plaque, are often found as oxysterols. Oxysterols are more bioactive than cholesterol, and their increased levels in human plaques correlate with disease severity [32]. Therefore, to establish a model of the oxysterol-rich foam cell that mimics the foam cells in atherosclerotic plaques, we first treated elicited mouse peritoneal macrophages (MPMs) with acetylated, moderately and highly-oxidized LDLs (acLDL, oxLDL, and hi-oxLDL, respectively) to assess the ratio of intracellular cholesterol and oxysterols. AcLDL-treated foam cells displayed larger LDs, visualized by Bodipy 483/503, than oxLDL-treated foam cells (Figure 1A green,B). Using a targeted sterol lipidomic analysis, we measured sterol and oxysterol contents in acLDL-, oxLDL-, and hi-oxLDL-treated MPMs. LDL loading increased total sterols in all three LDL treated conditions (Figure 1C left). In accordance with the knowledge that acLDL is a potent ACAT activator and in line with the larger LD size shown in Figure 1A,B, nearly 80% of sterols were esterified in acLDL-treated foam cells (Figure 1C middle). This was two times higher than that of oxLDL-treated foam cells (Figure 1C middle) [39,40]. However, when it comes to oxysterols, acLDL did not increase the cellular oxysterol level but it rather slightly reduced total oxysterols (Figure 1C left). In oxLDL-treated foam cells, we found an increased abundance of oxysterols in proportion to the degree of oxidation; oxysterols were 5 and 10 times higher in oxLDL-and hi-oxLDL-treated foam cells, respectively than in untreated controls (Figure 1C left) [39]. In addition, these oxysterols are esterified as seen in human plaques (Figure 1C. right) [41]. Figure 1 data show that hi-oxLDL-loaded foam cells contain the highest oxysterol level, and therefore, they are a better model system than acLDL-treated foam cells to study oxysterol dynamics mediated by cholesterol acceptors.

**Figure 2:**
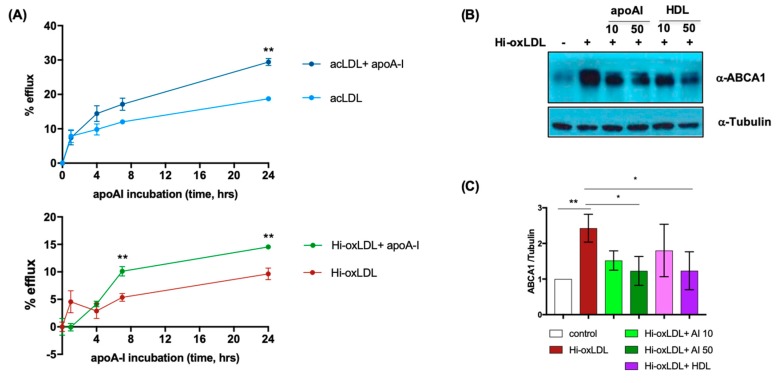
ApoA-I increases efflux of free cholesterol (FC) from hi-oxLDL-loaded foam cells. (**A**) MPMs were loaded with ^3^H-cholesterol-coupled acLDL or hi-oxLDL. Cholesterol efflux was initiated with 50 μg/mL of apoA-I. The cells without apoA-I loading were used as a control. The net percentage of cholesterol efflux at each time point compared to time 0 was presented as % efflux (*n* = 4 per each treatment). (**B**,**C**) Hi-oxLDL-loaded MPMs were loaded with 10 or 50 μg/mL of apoA-I or HDL. Immunoblotting with anti-ABCA1 and anti-tubulin were performed, and the relative level of ABCA1 was normalized with tubulin and quantified (*n* = 3) using Fiji software. The data for both cholesterol efflux and WB were presented as mean ± SEM. * *p* < 0.05, ** *p* < 0.005.

Next, we tested whether cholesterol efflux from hi-oxLDL-loaded foam cells is also mediated by cholesterol acceptors as seen in acLDL-and oxLDL-loaded foam cells [42,43]. We performed cholesterol efflux assays using ^3^H-cholesterol-labeled acLDL as the control or ^3^H-cholesterol-labeled hi-oxLDL followed by incubation with apoA-I. As known, there was passive cholesterol diffusion in acLDL- and hi-oxLDL-loaded foam cells in the absence of cholesterol acceptors [44]. Stimulation of these foam cells with apoA-I enhanced cholesterol efflux from hi-oxLDL-loaded foam cells like in acLDL-loaded foam cells (Figure 2A). HDL also enhanced cholesterol efflux from both acLDL- and hi-oxLDL-loaded foam cells (Figure S1). Intracellular free sterols, such as cholesterol and oxysterols, activate LXRs, resulting in increased expression of cholesterol transporters (e.g., ABCA1) to facilitate cholesterol efflux [45]. We also found that cells treated with hi-oxLDL had increased levels of ABCA1; conversely, treating cells with apoA-I or HDL decreased ABCA1, indicating possible clearance of intracellular free sterols (Figure 2B,C). Therefore, to mimic the oxysterol-enriched foam cells in the atherosclerotic plaques, we treated MPMs with hi-oxLDL to investigate the foam cell lipidome regulated by cholesterol acceptors in the following experiments.

### 2.2. Lipid-Poor apoA-I Significantly Reduces Oxysterols

**Figure 3:**
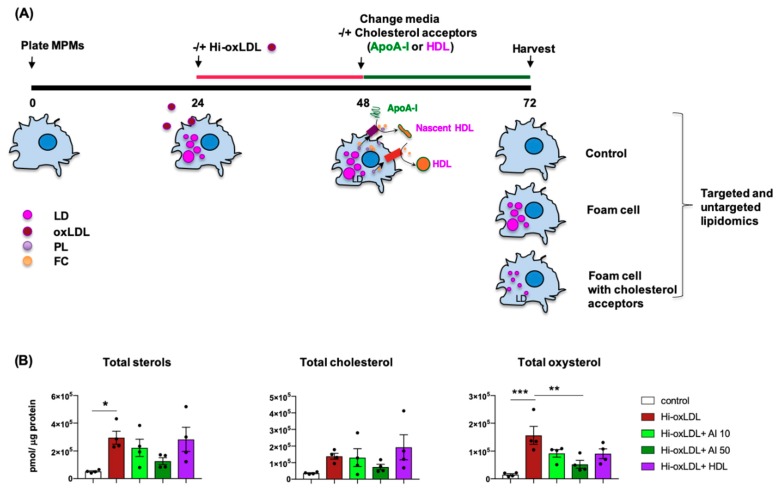
ApoA-I significantly reduces total sterols, cholesterol, and oxysterols in foam cells. (**A**) Experimental procedure. MPMs were loaded with hi-oxLDL (50 μg/mL) for 24 h. The media was refreshed without hi-oxLDL followed by incubation with or without apoA-I (10 μg/mL and 50 μg/mL) or HDL (50 μg/mL) for 24 h. Lipids were extracted, and sterol species were identified by MS. (**B**) The levels of total sterols, cholesterol, and oxysterols were presented as abundance obtained as pmol of lipids normalized with μg of protein. The data were presented as mean ± SEM (*n* = 4 per group). * *p* < 0.05, ** *p* < 0.005. *** *p* < 0.0005.

Cholesterol efflux from foam cells to cholesterol acceptors is well-established as the first step of RCT, which prevents and reverses atherogenesis [23]. Recent advances in lipid detection such as mass-spectrometry (MS) based lipidomics revealed the complexity of lipid species in human plasma and atherosclerotic plaques [31,46,47]. Although cholesterol is a major form of sterol in atherosclerotic lesions and plasma LDL, it is now known that abundant cholesterol precursors and derivatives are also detected in human plaques [47]. Notably, oxysterol concentrations normalized to cholesterol were 43 times higher in human carotid plaques compared to the plasma [47]. Therefore, first, we tested how apoA-I and HDL impact various sterol levels in foam cells using targeted sterol lipidomics. As depicted in Figure 3A, hi-oxLDL-induced foam cells were washed and treated with or without apoA-I (10 and 50 μg/mL) or HDL (50 μg/mL). Lipids were extracted and the sterols were identified using MS as detailed in the method section. Hi-oxLDL increased total detectable sterols about 5.7-fold and intracellular oxysterols up to 10-fold compared to untreated macrophages (control) (Figure 3B). Both doses of apoA-I (10 and 50 μg/mL) decreased total sterols, cholesterol, and oxysterols (Figure 3B). However, a statistically significant reduction was only accomplished by 50 μg/mL of apoA-I (Figure 3B). Interestingly, HDL treatment exerted a trend toward reduced total sterols, cholesterol, and oxysterols, but the degree of reduction was not significant possibly due to large variation among the samples in the group.

**Figure 4:**
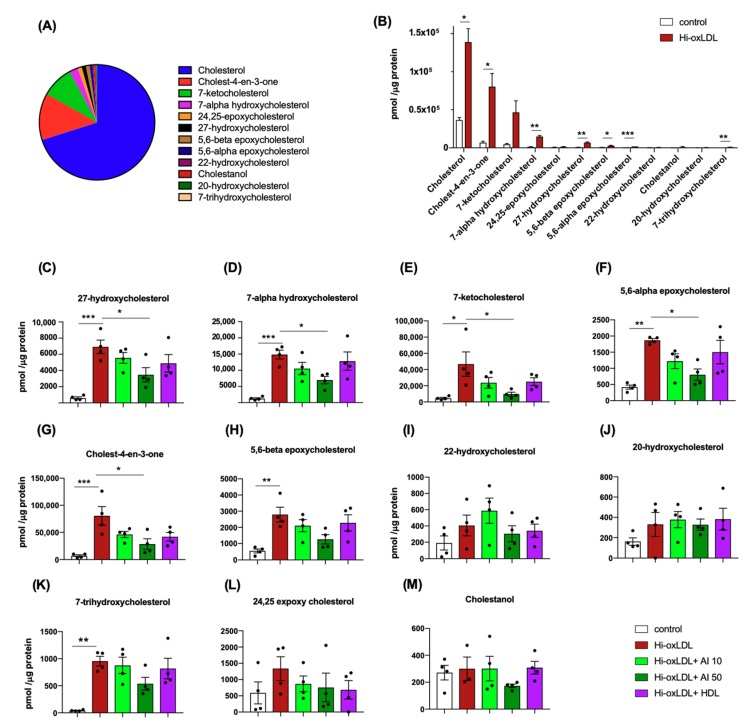
Targeted sterol lipidomics. (**A**) Relative levels of 12 sterols were presented based on their % of abundance to total sterols detected in untreated MPMs. (**B**) Increased levels of oxysterols upon hi-oxLDL loading were presented as abundance (**C–M**) Abundance of individual sterol species with apoA-I and HDL treatments. The abundance of lipids in (**B**–**M**) was calculated by pmol of lipid normalized to μg of protein. The data were presented as mean ± SEM (*n* = 4 per group). * *p* < 0.05, ** *p* < 0.05, *** *p* < 0.0005.

We next analyzed which sterol compounds are affected by cholesterol acceptors. Without any treatment, MPM’s sterols were composed of cholesterol (70%), cholest-4-en-3-one (12.6%), 7-ketocholesterol (8.9%), 7-α-hydroxycholesterol (2.4%) and others (Figure 4A). In hi-oxLDL-treated foam cells, most of these sterol species were significantly elevated compared to the sterols in the untreated control group (Figure 4B,C–H,K). Among those sterols, cholesterol, 7-ketocholesterol, 7-α-hydroxycholesterol, 27-hydroxycholesterol, 5,6-α-epoxycholesterol and 5,6-β-epoxycholesterol were found in human carotid plaques [47].

Accumulation of sterols oxidized at the 7^th^ carbon of the B ring such as 7-ketocholesterol and 7-α-hydroxycholesterol is associated with ER stress, macrophage apoptosis, and plaque necrosis [48,49]. 27-hydroxycholesterol was shown to enhance atherogenesis via increasing inflammatory gene expression, and it was abundantly found in complex and ulcerated human aorta samples [32,34]. Individual sterol compounds significantly down-regulated by apoA-I (50 μg/mL) were 27-hydroxycholesterol, 7-α-hydroxycholesterol, 7-ketocholesterol, 5,6-α-epoxycholesterol, and cholest-4-en-3-one (Figure 4C–G). The sterol species that remained unchanged were 5,6-β-epoxycholesterol, 20- and 22-hydroxycholesterols, 7-trihydroxycholesterol, 24,25-expoxycholesterol, and cholestanol (Figure 4H–M). Therefore, the sterol panel shows cholesterol acceptors, especially the high dose of lipid-poor apoA-I, significantly reduced pro-inflammatory and pro-atherogenic oxysterols in foam cells.

### 2.3. Major Lipid Categories and Lipid Species Linked to Atherogenesis are Down-Regulated by Cholesterol Acceptors

**Figure 5:**
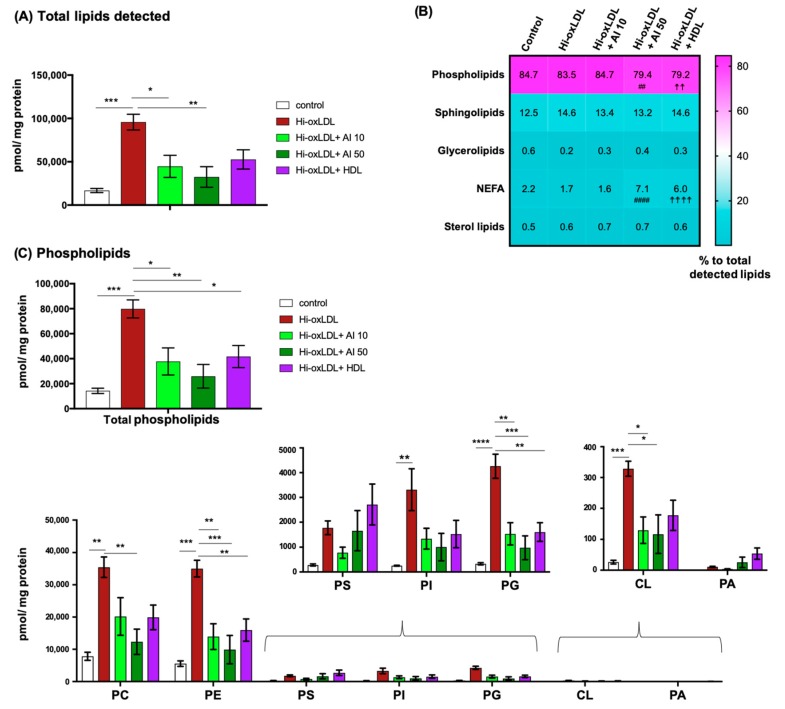

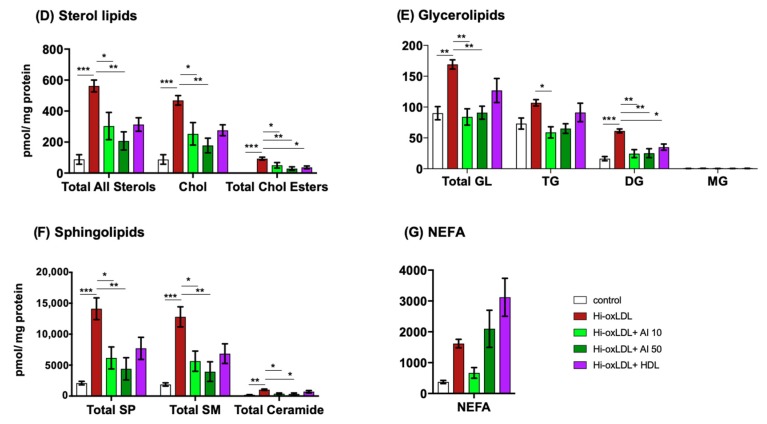
Untargeted global lipidomics. (**A**) Total lipids detected in each treatment group. (**B**) Heat map showing the relative abundance of five lipid categories as % of each category to total detected lipids. (**C**) Phospholipids. Total amount of phospholipids detected in each treatment group (top) and each class of phospholipids regulated by cholesterol acceptors (bottom). (**D**–**G**) The levels of sterol lipids, glycerolipids, sphingolipids, and non-esterified fatty acids (NEFA) in each group. The amount of lipid detected was presented as pmol lipid normalized to mg protein. TG; triglyceride, DG; diglyceride, MA; monoglyceride. The data were presented as mean ± SEM (*n* = 4 per group). * *p* < 0.05, ** *p* < 0.005, *** *p* < 0.0005, **** *p* < 0.00005. Hi-oxLDL versus hi-oxLDL + A-I 50: ^##^ *p* < 0.005, ^####^ *p* < 0.00005. Hi-oxLDL versus hi-oxLDL+ HDL: ^☨☨^ *p* < 0.005, ^☨☨☨☨^ *p* < 0.0005.

The cellular lipidome is composed of five major lipid categories, namely phospholipids (PL), fatty acyls (FA), glycerolipids (GL), sphingolipids (SP), and sterol lipids (ST) [50]. To gain insights into distributions of these five lipid categories in the foam cells, we performed untargeted global lipidomics. Hi-oxLDL-loaded MPMs exhibited an increase in total detectable lipids about 5.7-fold compared to untreated control MPMs (Figure 5A). All three conditions of acceptor treatments reduced the total amount of lipids in foam cells (Figure 5A). Again, the most significant reduction was exerted by apoA-I at 50 μg/mL. We analyzed whether the reduction of total lipid contents by apoAI and HDL leads to the redistribution of lipid categories or not. As shown in the Heatmap analysis, lipids in untreated MPMs consisted of 84.7% PL, 12.5% SP, 0.6% GL, 2.2% non-esterified fatty acids (NEFA), and 0.5% ST (Figure 5B, first column). Hi-oxLDL loading increased the abundance of all five categories of lipids (Figure 5C–G). Compared to the hi-oxLDL-treated group, apoA-I and HDL treatments at 50 μg/mL reduced the abundance of PL as well as their relative ratio to total lipid contents (Figure 5B, fourth and fifth column and 5C) confirming efficient PL efflux by cholesterol acceptors as reported previously [51]. Relative % of NEFA to total detected lipids without significant changes of the amount (Figure 5G) were higher with apoA-I and HDL treatments at 50 μg/mL compared to the hi-oxLDL-treated group, suggesting cholesterol acceptors do not mediate NEFA transport. 

Next, we analyzed how the subclasses of each lipid category were affected. PL is a major component of the lipid monolayer of lipoproteins. PL is also the most abundant lipid category and the main component of the plasma membrane (PM) and membranes of the intracellular organelles [29,52,53]. Hi-oxLDL-induced foam cells contained 4 times more total PL than untreated control cells (Figure 5C top). These increases occurred in the all classes of PL including phosphatidylcholine (PC), phosphatidylethanolamine (PE), phosphatidylserine (PS), phosphatidylinositol (PI), phosphatidylglycerol (PG), cardiolipin (CL), and phosphatidic acid (PA) (Figure 5C bottom).

Among these subclasses, PC, PE, PG and CL were significantly reduced by apoA-I (Figure 5C bottom). PC and PE are the first and second most abundant PL classes, respectively, in the membranes of cellular organelles and lipoproteins, and PC is effluxed together with FC via ABCA1 to apoA-I [51,54]. PG is an intermediate lipid of CL synthesis in mitochondria, and CL is exclusively localized in inner membrane of mitochondria. CL is involved in important mitochondrial functions such as oxidative phosphorylation, mitophagy, and mitochondrial apoptosis [55]. CL is required for NLRP3 inflammasome activation when macrophages are stimulated by LPS, and cholesterol crystals in the atherosclerotic lesion induce inflammations through NLRP3 inflammasomes activation [56,57]. This suggests CL can act as mediator of inflammation, and clearing CL might represent an important and yet poorly understood anti-inflammatory action of apoA-I [58,59].

LDL carries CE and triglyceride (TG) in its core [30]. After internalization of LDL into macrophages, in the lysosome, these esterified lipids are hydrolyzed into un-esterified lipids such as FC and free fatty acid (FA). Excess amounts of these free form of lipids are either effluxed or re-esterified in the ER followed by immediate incorporation to the core of LDs to avoid cytotoxicity [60,61,62]. As seen in the sterol panel analysis in Figure 3, global lipidomics also found a significant elevation in total sterol levels with hi-oxLDL treatments and their levels are considerably reduced with apoA-I incubation whether they are free or esterified forms (Figure 5D). Total GL, which consists of monoglycerides (MG), diglycerides (DG), and TG, was two times higher in the hi-oxLDL-induced foam cells (Figure 5E). MG was almost undetectable in both control and foam cells. Surprisingly, the levels of both TG and DG were reduced to basal levels by apoA-I treatments, but HDL did not affect the TG level whereas DG were down regulated by all three-acceptor treatments (Figure 5E). TG are rich in LDs whereas DG play as second messengers in the PM and are intermediates of other subclasses of PL [63]. These results suggest that apoA-I, but not HDL, regulates TG mobilization from LDs.

Hi-oxLDL loading also increased total SP (6.7-fold) and its subclasses, sphingomyelin (SM, 6.82-fold) and ceramides (5.12-fold). Ceramides are synthesized in the ER and transported to Golgi apparatus where SM is generated. As reviewed by Bismuth et al., multiple lines of evidences showed that accumulated SP in atherosclerotic lesion regulated in lesional lipoprotein metabolism resulting in increased atherosclerosis [64]. Significant reductions in SP and SM were observed in apoA-I treatment groups (Figure 5F). Some SM species including SM(d18:1/16:0), SM(d18:1/24:1), SM(d18:1/24:2), SM(d18:1/24:0), SM(d18:15:0), SM(d18:1/16:1), and SM(d18:1/14:0) are specifically detectable in human carotid plaques compared to radial arteries [31]. SM(d18:1/16:0), SM(d18:1/24:1), SM(d18:1/24:2), and SM(d18:1/24:0) are the top 4 most abundant SMs in our lipidomics study, and their levels are down-regulated by cholesterol acceptors (Figure S2 and Table S1). SM is abundant in the lysosome and PM of cells and lipoproteins [46]. SM and cholesterol are also abundant in lipid rafts, which are transient, ordered, and detergent resistant microdomains of PM [65]. Depletion of SM in the PM increased cholesterol efflux by increasing ABCA1 and ABCG1 [66]. Therefore, enriched SM levels in foam cells and human plaque might contribute to defective cholesterol efflux, and clearing SM by cholesterol acceptors might lead to increasing cholesterol removal thereby reducing foam cells. In peritoneal foam cells of LDL receptor knock out mice (LDLR KO), it was reported that increased NEFA was produced by the hydrolysis of TG from LDL. Major FAs in this study were palmitic acid, 9z-palmitoileic acid and oleic acid, which are found in CE, PG and TG [58]. In our study, we also observed elevated NEFA levels in hi-oxLDL-induced foam cells. Stearic acid (36.6%), palmitic acid (14.4%), and oleic acid (11%) were the most abundant FA species. Esterification of FA into cholesterol generates CE. As reported oleic acid is a major FA found in CE, and we also found the dominant FAs incorporated into CE were oleic acid (CE 18:1, 43.7%) and linoleic acid (CE 18:2, 18.3%). These two CEs were also the most abundant CE species in human endarectomy specimens [67]. Additionally, CE 18:0 was significantly elevated in unstable human plaques [67]. Our lipidomics found there was no detectable CE 18:0 in control MPMs. But its levels were increased with hi-oxLDL loading, and cholesterol acceptors reduced CE 18:0 levels. Although cholesterol acceptors significantly reduced these CE species, they did not significantly alter the level of NEFA, indicating cholesterol acceptors are more actively involved in cholesterol efflux rather than FA efflux.

## 3. Discussion

Antiatherogenic mechanisms of apoA-I and HDL have been extensively studied [68]. In addition to promoting macrophage cholesterol efflux, these particles have been shown to exert pleiotropic protective effects, including modulation of innate immune response, recruitment and proliferation of inflammatory cells, inflammation, efferocytosis, etc. However, despite their recognized roles as extracellular lipid acceptors, little is known about their overall effects on foam cell lipidomes, and whether the athero-protective properties of these particles also include removal of pro-atherogenic lipid mediators.

In this study, we performed lipid profiling of hi-oxLDL-induced macrophage foam cells and investigated the influence of cholesterol acceptors on this lipid profile. Foam cells are also formed by transdifferentiated smooth muscle cells (SMCs) of the lesion [11,12]. However, the mechanistic studies in lipid trafficking in SMC originated foam cells remain elusive. Therefore, we used macrophages to study how cholesterol acceptors handle foam cell lipidomes. Targeted (for sterols) and untargeted lipidomics detected a total of 12 and 504 lipid compounds, respectively. Similar to human plaque lipid compositions, our targeted lipidomics in hi-oxLDL-induced foam cells detected cholesterol as the most abundant sterol, and a significant increase of 8 oxysterols including cholest-4-en-3-one, 7-keto and 7-α-hydroxycholesterols, and 27-hydroxycholesterol that have been implicated in the pathogenesis of atherosclerosis [34,49]. In addition to elevated ST in hi-oxLDL-loaded foam cells, our untargeted global lipidomics also discovered substantial increases in four other lipid categories: PL, SP, GL, and NEFA. Unexpectedly, instead of reducing only PL and cholesterol, stimulating foam cells with cholesterol acceptors induced significant decreases in total lipid contents across the four major categories of lipid species except NEFA, suggesting that extracellular acceptors have the ability to reverse the overall impact of oxLDL loading. In general, the higher dose of apoA-I brought about the most distinctive effect on the foam cell lipidome. Analyses on individual compounds found that among the ST, the five most abundant oxysterols in human plaques were significantly reduced by apoA-I. In addition, non-sterol lipid compounds recently identified in human plaques, such as SM and ceramides, are remarkably reduced by apoA-I. A possible reason HDL exerted less potent effects on lipidome regulation than apoA-I is because HDL is already filled with lipids compared to its lipid-poor precursor, apoA-I, and it utilizes a different transporter, ABCG1, in the PM while apoA-I uses ABCA1 [69].

In vitro foam cells formed by modified-LDL loading in cultured macrophages brought contrary results in expression of inflammatory genes [70,71,72,73]. Given the complexity of foam cell microenvironments in the atheroma, how lipid loading affects inflammation needs to be investigated within the context of a given microenvironment during atherogenesis [13,74,75]. Recently, transcriptomics of thioglycolate-elicited foam cells from LDLR KO mice fed with a Western diet (WD) identified down-regulated inflammatory gene expression [58]. Our previous transcriptome analysis of foam cells isolated from the lesion of apo E KO mice found insignificant changes in most inflammatory gene expression in fully developed lesions compared to early lesions of WD-fed apoE KO mice [76]. In addition, single cell RNA-seq analysis from the mouse atherosclerotic lesion also identified reduced inflammatory gene expression in ten different subgroups of foamy leukocytes [59]. These studies indicate foam cell formation is beneficial to reduce lesional inflammation. However, a supplement of mixed oxysterols in WD-fed apoE KO and LDLR KO mice was reported to promote atherogenesis [33,77]. Additionally, an elevation of 27-hydroxycholesterol in cyp7b1 KO/apoE KO mice exacerbated atherosclerosis and subcutaneous injection with 27-hydroxycholesterol to apoE KO mice increased atherosclerosis [34]. These studies suggest that despite the protective effect of foam cells from inflammation, elevation of certain oxysterols in foam cells adversely regulates atherosclerosis. Moreover, multiple studies found increased abundance in cytotoxic oxysterols and non-sterol lipid species in human advanced, unstable and symptomatic plaques, suggesting a role of these lipid species in plaque vulnerability [32,47,64,78]. It is possible that continuous influx of oxLDL to foam cells outpaces maximum ACAT activity and leads to accumulation of free sterols in cellular membranes. Free oxysterols induce apoptosis and defective efferocytosis, and in a scenario in which dying macrophage/foam cells spill their lipid contents in the lesion, it may generate a pro-thrombotic necrotic core [74]. It remains unclear how lipidomes of lesional foam cells are dynamically altered in different stages of atherosclerosis and among the diversity of foam cells. Along with development of more sensitive lipidomic analyses, many more studies are required to understand the metabolism of individual lipid species, especially lipids enriched in the human atheroma, in the foam cells and its impact on lipid-mediated signaling cascades in the context of the plaque microenvironment. In addition to inter-individual variability, ratios of lipid content within the same plaque can be highly variable, indicating complex dynamics in lipid metabolism that might challenge the interpretation of these analyses [78]. The intrinsic relationship between atherosclerosis and lipid metabolism warrants interest in untangling the complex interrelationship between lipids and other major components in atherosclerosis, for example their role in the regulation of vascular inflammation.

Information missing in this study is a spatial distribution of the lipidome, which can be revealed by lipidomics on isolated PM and intracellular organelles. In addition, our study does not elucidate whether non-sterol lipid species are effluxed to apoA-I/ HDL or catabolized inside cells upon apoA-I/ HDL stimulation. It is still an open question of how some lipids predominantly found in certain intracellular organelles (e.g., mitochondria or lysosomes) are reduced by extracellular apoAI or HDL, and whether ABCA1 or ABCG1 also mediates the transport of non-sterol lipids as they do for PL. In search of the lipid transporter, proteome analyses of PM and intracellular organelles in the absence and presence of apoA-I and HDL would provide preliminary information about the details of this trafficking. In addition, it could be worthwhile to perform lipidomics in the absence of known PM transporters to determine the differential mechanisms by which apoA-I and HDL regulate the foam cell lipidome along with lipidomics of isolated intracellular organelles and PM.

In conclusion, the unique features of our study include, firstly, the comparison of over 500 lipid species between untreated macrophages and hi-oxLDL-induced foam cells, which model the oxysterol rich-lipidome of human macrophage foam cells in atheromas. Secondly, we identified cholesterol acceptors, especially apoA-I, as inverse regulators of various pro-atherogenic lipid species. Overall, our study re-enforces the positive role of the cholesterol acceptors, especially apoA-I, as potential targets to ameliorate atherosclerosis due to its strong ability to reprogram the lipidome of foam cells beyond its effects on FC. Our findings stress the need to develop a local delivery system to facilitate apoA-I’s access to foam cells within lesions as a key strategy to prevent atheroma growth and reverse plaque size and increase plaque stability.

## 4. Materials and Methods

### 4.1. Reagents

Bodipy 493/503 is from Invitrogen and DAPI (4′,6-diamidino-2-phenylindole) is from Sigma (St. Louis, MO, USA). Anti-ABCA-1 and anti-beta-tubulin antibodies were purchased from Novus Biologicals (Centennial, CO, USA). Human acLDL (J65029), oxLDL (J65591), hi-oxLDL (J65261), apoA-I (J64506) and HDL (64903) are from Alfa Aesar (Haverhill, MA, USA). [(1α,2α(N)-^3^H] cholesterol is from Perkin Elmer (Waltham, MA, USA). ProLong Antifade Mountant was purchased from ThermoFisher (Agawam, MA, USA).

### 4.2. Mice

Wild-type C57BL/6J male mice aged 8–12 weeks were obtained from Jackson Laboratory (stock #000664). According to animal welfare guidelines, mice were housed under 12 h of light and dark cycles and had full access to regular rodent chow diet and water during the entire study. At the time of experiments, age of mice was about 11–12 weeks. All procedures on mice, conducted in this study, were approved by the Institutional Animal Care and Use Committee at Albany Medical College to principal investigator: Goo, Young-Hwa, Protocol number: 17-06002, date of protocol approval for continuing use from 19 September 2018 to 19 September 2019.

### 4.3. Cell Culture

Mouse peritoneal macrophages (MPMs) from the peritoneal cavity were harvested 5 days after aged 3% thioglycolate injection into C57BL/6J mice. MPMs (4–5 × 10^6^ cells per dish) were plated in 60mm culture dishes with RPMI 1640 supplemented with 10% FBS. A day later, to generate foam cells, MPMs were treated with various LDLs as described in the figure legends. For the study of lipidome regulation by cholesterol acceptors, one day after LDL loading the media was switched to RPMI 1640 containing 10% FBS to remove un-internalized LDLs. Then the cells were incubated in the absence or presence of apoA-I (10 and 50 μg/mL) or HDL (50 μg/mL) for 24 h as illustrated in Figure 3. The cells were harvested with cold 1× PBS and the pellets were stored at −80 °C until lipidomic analyses.

### 4.4. Lipid Droplet Staining and Measuring LD Area

MPMs were cultured on a coverslip for 1 day and treated with acLDL (50 μg/mL) or Hi-oxLDL acLDL (50 μg/mL) for 24h (*n* = 3 per treatment). Untreated MPMs were used as a control. The cells were fixed with 3.7% P-formaldehyde for 15min at R.T. After washing with 1× PBS three times, the cells were stained with Bodipy 493/503 (5–10 μg/mL) to visualize lipid droplets (green) and DAPI (0.5–1 μg/mL) for nuclei staining (blue) at R.T for 30min. Cells were mounted with ProLong antifade mountant. Images were taken with Zeiss Observer D1 microscope and Cytation 5 imaging reader (BioTek, Winooski, VT, USA). Pictures of three randomly chosen areas per coverslip were analyzed with Gen5 3.05 software (BioTek, Winooski, VT, USA). Each area contained 50–70 cells. Total area of LDs and total fluorescence intensities per cells were obtained using Gen5 3.05 software.

### 4.5. Cholesterol Efflux

About 2.75 × 10^5^ MPMs were plated in a 24-well cell culture plate. One day later, the cells were incubated in 0.2% BSA/DMEM supplemented with acLDL or hi-TBAR oxLDL (50 μg/mL) labeled with [1α,2α(N)-^3^H] cholesterol. After 24 h, MPMs were thoroughly washed with 1× PBS and equilibrated in 0.2% BSA/DMEM for 1 h. Then MPMs were incubated with or without apoA-I (50 μg/mL) or HDL (50 μg/mL) for 24 h. Aliquots of media were collected at 0, 1, 4, 8 and 24 h after apoA-I or HDL loading. At the final time point, the cells were lysed in 0.1N NaOH. The radioactivity in the media and cells was determined by scintillation counting. The net percentage of cholesterol efflux at each time point was calculated by subtracting cpm value of time zero from the cpm of each time point.

### 4.6. Lipid Extraction

Approximately 4–5 × 10^6^ MPMs were subjected to monophasic lipid extraction in methanol:chloroform:water (2:1:0.74, *v*:*v*:*v*) as previously described [46,79]. Pelleted cellular proteins were subsequently dissolved in 0.1 N NaOH to determine the total amount of protein per sample group and used for normalization of lipids detected. Di-myristoyl phosphatidylcholine (Avanti Polar Lipids, Alabaster, AL, USA) and 19-hydroxycholesterol (Steraloids, Newport, RI, USA) were added to samples during lipid extraction at 1 nmol/mg protein as an internal standard for relative quantitation of lipids and sterols. Dried lipid extracts were desalted by washing as previously described and resuspended using 200 µL/mg protein in a solution of methanol containing 0.01% butylated hydroxytoluene [79]. The samples were stored under a blanket of nitrogen at −80 °C until further analysis.

### 4.7. Global Lipidomics Analysis

For each analysis, lipid extracts were diluted into isopropanol: methanol (2:1, *v*:*v*) containing 20 mM ammonium formate and analyzed by flow injection high resolution/accurate MS and tandem MS as previously described [80].

### 4.8. Analysis of Free and Total Sterol Content

Sterols and oxysterols were analyzed by high resolution/accurate mass LC-MS using a Shimadzu Prominence HPLC coupled to a Thermo Scientific LTQ-Orbitrap Velos mass spectrometer as previously described [81]

### 4.9. Peak Finding, Analyte Identification, and Quantification

For global lipidomics experiments, lipid species were identified using the Lipid Mass Spectrum Analysis (LIMSA) v.1.0 software linear fit algorithm, in conjunction with a user-defined database of hypothetical lipid compounds for automated peak finding and correction of ^13^C isotope effects. Relative quantification of lipid abundance between samples was performed by normalization of target lipid ion peak areas to the di-myristoyl phosphatidylcholine internal standard as previously described [82]. For free and total sterol analysis, chromatographic peak alignment, compound identification, and relative quantitation against the 19-hydroxycholesterol internal standard were performed using MAVEN software 3.6.1 [83].

### 4.10. Data Analysis

For multiple comparison data analysis, we performed one-way ANOVA with Turkey’s multiple comparison for adjusted *p* values. Comparison between two groups in the Figure 1B right, Figure 2A, Figure 4B, and Figure S1 were performed using Student’s t-test. All error bars represent as SEM.

## Supplementary Materials

Supplementary materials can be found at https://www.mdpi.com/1422-0067/20/15/3784/s1.

## Abbreviations

HDL	High-density lipoprotein
ApoA-I	apolipoprotein A-I
CE	Cholesterol ester
LDL	Low-density lipoprotein
PL	Phospholipids
GL	Glycerolipids
SL	Sphingolipids
ST	Sterol lipids
NEFA	Non-esterified fatty acids
SM	Sphingomyelins
ABCA1	ATP-binding cassette transporter member 1
ABCG1	ATP-binding cassette subfamily member G1
OxLDL	Oxidized LDL
AcLDL	Acetylated LDL
MS	Mass-Spectrometry
MPM	Mouse peritoneal macrophage
PM	Plasma membrane
