# Supplementary material for: Cholesterol Acceptors Regulate the Lipidome of Macrophage Foam Cells
Source: Int J Mol Sci. 2019 Aug 2;20(15):3784. doi: 10.3390/ijms20153784 (PMC6695943; doi:10.3390/ijms20153784)
Supplement: Supplementary file 1 [file ijms-20-03784-s001.pdf]

Supplementary Materials:

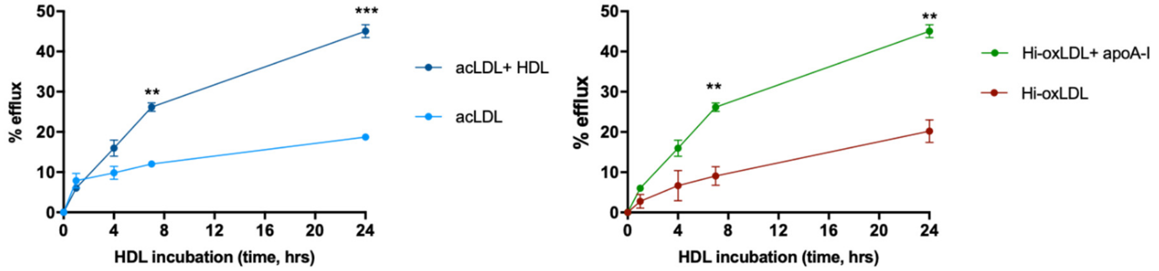

Figure S1. MPMs were loaded with ^{3}H-cholesterol-coupled acLDL or hi-oxLDL. Cholesterol efflux was initiated with 50 µg/mL of HDL. The percentage of cholesterol efflux compared to time 0 at each time point was calculated as described in the methods (*n* = 4 per each treatment). The data were presented as mean ± SEM. $^{**}p < 0.005$, $^{***}p < 0.0005$.

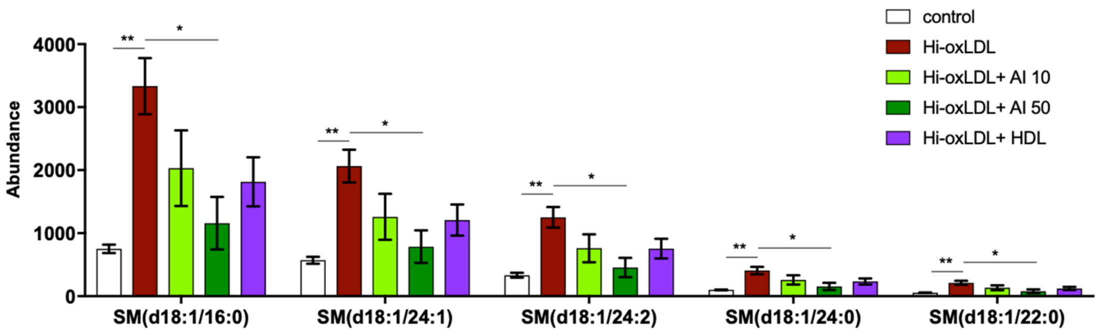

Figure S2. Top five most abundant sphingomyelin (SM) compounds in each treatment group. The values are ion abundance normalized to mg protein. The data were presented as mean ± SEM (*n* = 4 per group). Control versus hi-oxLDL, $^{*}p < 0.05$, $^{**}p < 0.005$.

Table S1. All sphingomyelin (SM) compounds detected from untargeted lipidomics. SMs that were found in human endarterectomy but not in the radial artery [31] were colored in red. (*n* = 4 per group).

		**Control**		**Hi-oxLDL**		**Hi-oxLDL + AI 10**		**Hi-oxLDL + AI 50**		**Hi-oxLDL + HDL 50**	
**m/z**	**Lipid**	Average	SEM	Average	SEM	Average	SEM	Average	SEM	Average	SEM
703.5748124	**SM(d18:1/16:0)**	752.53	65.91	3333.84	445.84	2031.61	600.97	1159.25	417.20	1813.97	389.87
813.6843568	**SM(d18:1/24:1)**	570.68	54.18	2065.40	260.37	1259.39	363.98	785.51	258.47	1208.92	246.02
811.6687076	**SM(d18:1/24:2)**	332.69	37.63	1251.40	163.33	761.02	222.23	454.41	153.45	756.00	156.08
815.700006	**SM(d18:1/24:0)**	100.31	6.02	407.88	58.61	258.61	74.43	154.70	55.49	234.23	47.76
787.6687076	SM(d18:1/22:0)	55.11	3.08	212.80	30.84	134.20	38.96	79.93	28.45	123.28	25.55
725.5591632	SM(d18:1/18:3)	8.81	1.56	74.35	15.71	41.61	12.74	20.12	8.01	33.28	8.59
689.5591632	**SM(d18:1/15:0)**	11.37	1.25	60.80	8.90	36.87	11.10	20.16	7.56	30.12	6.45
799.6687076	SM(d18:1/23:1)	18.86	1.74	58.45	7.69	35.32	10.15	22.63	7.60	31.81	6.44
801.6843568	SM(d18:1/23:0)	12.77	0.78	57.68	8.41	35.58	10.27	21.39	7.44	33.19	6.72
731.6061108	SM(d18:1/18:0)	12.10	0.88	53.68	7.51	33.38	10.06	19.30	7.23	30.69	6.67
701.5591632	**SM(d18:1/16:1)**	9.05	1.23	46.66	6.19	27.15	8.29	16.57	5.69	23.08	4.96
785.6530584	SM(d18:1/22:1)	12.06	1.17	43.39	5.77	27.07	7.92	17.82	5.72	23.78	4.88
809.6530584	SM(d18:1/24:3)	11.06	1.27	41.11	5.06	24.30	7.09	14.90	4.91	23.32	4.98
675.543514	**SM(d18:1/14:0)**	4.42	0.61	30.82	4.71	18.27	5.59	9.90	3.97	14.71	3.25
717.5904616	SM(d18:1/17:0)	6.02	0.73	27.55	4.04	16.49	4.97	9.72	3.84	14.65	3.10
759.6374092	SM(d18:1/20:0)	3.62	0.48	17.92	2.46	10.93	3.33	6.41	2.47	10.24	2.09
783.6374092	SM(d18:1/22:2)	2.69	0.46	13.30	1.60	8.18	2.45	4.81	1.77	8.03	1.66
827.700006	SM(d18:1/25:1)	3.31	0.51	13.26	1.84	8.26	2.41	5.03	2.03	7.27	1.47
839.700006	SM(d18:1/26:2)	2.79	0.57	13.12	1.70	7.65	2.31	4.57	1.86	6.73	1.49
841.7156552	SM(d18:1/26:1)	1.26	0.43	6.70	1.34	3.82	1.13	2.13	1.03	2.93	0.53
797.6530584	SM(d18:1/23:2)	1.09	0.29	5.77	1.19	3.55	1.15	2.03	0.87	3.04	0.70

729.5904616	SM(d18:1/18:1)	0.40	0.11	5.06	0.95	2.61	0.86	1.42	0.53	2.19	0.49
829.7156552	SM(d18:1/25:0)	0.59	0.14	4.35	0.87	2.63	0.84	1.16	0.57	1.94	0.51
825.6843568	SM(d18:1/25:2)	0.92	0.34	4.03	0.92	2.49	0.81	1.24	0.55	1.86	0.36
773.6530584	SM(d18:1/21:0)	0.25	0.13	3.05	0.92	0.00	0.00	0.00	0.00	1.52	0.61
869.7469536	SM(d18:1/28:1)	0.00	0.00	0.00	0.00	0.00	0.00	5.79	2.17	0.00	0.00
